# Sensitization to rubber allergens among 1,162 patients tested with the Brazilian standard battery^⋆^

**DOI:** 10.1016/j.abd.2022.02.003

**Published:** 2022-11-12

**Authors:** Maria Antonieta Rios Scherrer, Erica Possa de Abreu, Vanessa Barreto Rocha

**Affiliations:** a Service of Dermatology, Hospital das Clínicas da Universidade Federal de Minas Gerais, Belo Horizonte, MG, Brazil; b Department of Internal Medicine, Faculty of Medicine, Universidade Federal de Minas Gerais (UFMG), Belo Horizonte, MG, Brazil

Dear Editor,

Widespread sensitization to rubber components is well known and may be caused by latex or synthetic rubber additives. Other products, such as insecticides, clothing, medicines, and paints have similar chemical additives. Latex primarily causes type I and rubber vulcanizers and antioxidants are responsible for type IV allergies.^1^, ^2^

The Brazilian Standard Battery (BPB, *Bateria Padrão Brasileira*) features rubber mixtures (carba, thiuram, mercapto, PPD) and other related allergens, such as hydroquinone, ethylenediamine, and paraphenylenediamine (PPDA). Hydroquinone is an antioxidant, rarely used in industry at present.^3^ Ethylenediamine dihydrochloride stabilizes steroid creams and latex but it is not tested in other standard series.^4^ PPDA belongs to the benzene group and can cross-react with PPD-mix (*N*-Isopropyl-*N*-phenyl Paraphenylenediamine [IPPD], *N*-cyclohexyl-*N*-phenyl-*p*-phenylenediamine,*N*,*N*-diphenyl-*p*-phenylenediamine).^5^

From October 2009 to October 2018, 1,162 patients with suspected allergic contact dermatitis were treated at the Dermatology Annex of Hospital das Clínicas. They were tested with the BPB (FDA-allergenic, RJ, Brazil) using Finn chambers® (Oy, Finland) and readings were made at 48 hs and 96 hs, following the criteria of the ICDRG (International Contact Dermatitis Research Group).^6^ Data on age, occupation, affected sites, history of atopy, and allergen positivity were collected in Excel® throughout these years. In this retrospective study, the data from the aforementioned worksheet were consulted and, when necessary, the medical records as well, to complement the necessary information.

A total of 120 patients (10.3%) tested positive for rubber allergens; of these, 98 (81.7%) were clinically relevant based on patient history and allergen exposure. The demographic characteristics were described according to the MOAHLFA index (Table 1, Table 2).

**Table 1:** MOAHLFA* index in patients with positivity to rubber allergens.

	n (%)
**M**	59 (49,2)
**O**	61 (50.8) (40,8% ‒ construction workers)
**A**	30 (25)
**H**	70 (50.8)
**L**	48 (40)
**F**	43 (34.2)
**A**	82 (68.3)

M, Male sex; O, Occupational Dermatitis; A, History of Atopy; H, Dermatitis of the hands; L, Dermatitis of the legs; F, Dermatitis of the face; A, age 40 years or older.

* Uter W, Schnuch A, Gefeller O, ESCD working group: European Surveillance System on Contact Allergies. Guidelines for the descriptive presentation and statistical analysis of contact allergy data. Contact Dermatitis. 2004;51:47-56.

The prevalence of positivity for rubber mixtures was: carba, 41.7%; thiuram, 30%; mercapto, 15%; and PPD, 5% (Table 2).

**Table 2:** Frequencies of sensitization to the tested substances for rubber allergy.

Total number of cases		
	n	%
PPD-mix	6	5
Hydroquinone	6	5
Mercapto-mix	18	15
Thiuram-mix	36	30
Ethylenediamine	4	3,3
PPDA	49	40.8 (33 – 27.5% related to hair dyes)
Polysensitization > 3 allergens	39	32.5

An association between rubber allergens (cross-reactions) was observed in 35.8% of the cases: carbamates with thiurams in 16.6% and mercaptobenzothiazole with carbamates in 1.7%. Furthermore, the association between carbamates, mercapto and thiuram was observed in 8.3% of patients (Table 3).

**Table 3:** Associations of rubber mixtures with test positivity for each tested type.

Mixture	Thiuram	Mercapto*	PPD	PPDA+	Mercapto + Thiuram
Carbamates	20 / 120	2/120	5/120	0	10 / 120
	16.6%	1.70%	4.20%	0	8.30%
	Carbamates	Mercapto	PPD	PPDA	
Thiuram	0	0	0	3/120	
	0	0	0	2.50%	
	Mercapto	Thiuram	Carbamates	PPDA	
PPD				5/120	
				4.20%	
	Thiuram	Carbamates	PPD	PPDA	
Mercapto	0	0	0	1/120	
	0	0	0	0.80%	

+PPDA, Paraphenylenediamine; *Mercapto, Mercaptobenzothiazole; PPD (mix of N-isopropil-N-fenil parafenilenodiamina, N-ciclohexil-N-fenil parafenilenodiamina, N, N-difenil parafenilenodiamina).

Conde-Salazar et al. reported 14.7% of sensitization to rubber additives in 4,680 tested patients.^1^ Holness and Nethercott tested 1,670 patients, with 8.9% positivity to at least one rubber allergen, similar to what was found in the present study (10.2%).^7^ The ESSCA (European Surveillance System on Contact Allergies), when testing standard and rubber-specific batteries, found 8.8% of positivity. Other authors found between 3.8% and 15% of positive reactions to rubber components using standard batteries^8^ (Table 4).

**Table 4:** Comparison with other studies of positivity to rubber allergens.

Study	Number of patients	≥1 rubber allergen (%)	Standard series	Rubber-specific supplementary series
Conde-Salazar^1^	4.68	14.7%	Yes	Yes
Holness and Nethercott^7^	1.67	8.9%	Yes	Yes
Bendewald^2^	773	31.7%	Yes	Yes
Present study	1,162	10.3%	Yes	No

According to the MOHALFA index, the most affected location is the hands, both in the present and in other reports.^1^, ^2^, ^7^, ^9^ Atopy was present in 25% of the patients in the present report, in contrast to 4.2% in the study by Conde-Salazar et al.^1^

The main population affected by rubber sensitization are health and laboratory workers, followed by cleaning and construction workers, mainly due to the use of rubber gloves and boots. A high frequency of construction workers (40.8%) was detected in the present study, a fact also reported by Conde-Salazar et al. (47%).^1^ These authors showed greater positivity for thiuram (83%) in contrast to others who showed a decrease in thiuram and an increase in positivity for carbamates over the years. The present results confirmed this trend. Moreover, thiuram disulfides rarely appear in the final rubber product, although they can be used as additives.^1^, ^7^, ^10^

Healthcare professionals had a statistically increased risk of sensitization to carbamates and thiuram found in rubber gloves. Allergy to thiuram is also associated with domestic workers, restaurant workers, construction workers, and hairdressers.^6^, ^10^ In contrast, the present series showed a higher prevalence of carbamate sensitization, especially among construction workers.

Although carbamates are irritants and may cause false-positive reactions, some studies have indicated a true increase in their sensitization.^8^, ^9^

The BPB tests only the mercapto mixture, while others also test mercaptobenzothiazole, which is probably the true hapten present in this mixture. The data of the present study showed that its sensitization rate is similar to that of other studies.^1^, ^7^, ^8^

Some batteries do not use the PPD-mix, preferring IPPD, which is one of the components of the mix. However, some reports indicate an underdiagnosis of sensitization to this class of allergens.^8^ Its frequency was lower than the other substances in our study, a trend observed by other authors.^1^, ^6^, ^7^ Sensitization to PPD-mix is ​​significantly higher among industrial workers and hairdressers/barbers due to contact with para-amino agents present in permanent hair dyes.^10^ In the present study, PPDA showed 40.8% of sensitization, with 27.5% being related to hair dyes in women.

Conde-Salazar et al. found chromate sensitivity in 47% of construction workers allergic to rubber, whereas the present study found 40.8% and proposed that chromate would act as a strong sensitizer and irritant, facilitating greater sensitization to gloves.^1^

Positive associations among rubber additives are known.^1^ The present study showed 35.8% of these associations, with carba-thiuram being the most frequent one (16.6%), followed by carba-mercapto-thiuram (8.3%). During vulcanization, new compounds can be formed by reactions between carbamates, thiuram, and/or mercaptobenzothiazoles. Newly formed components can also cause sensitization; thus, it is recommended to test samples from gloves or boots to increase the sensitivity of patch tests.^9^

Hydroquinone monobenzyl ether can cause leukoderma, but its current use in industry is rare. Its prevalence in rubber-specific batteries was 0.82% in European patients. Ethylenediamine dihydrochloride showed lower positivity rates in 2,027 reported patients (0.69%).^9^ Removed from other series, they are present in the BPB and had a low prevalence in the present study (3.3% ethylenediamine and 5% hydroquinone).

In conclusion, there is currently an increase in awareness of carbamates at a global level. In addition, they are the most used accelerators in the industry, as the use of thiurams and mercaptos has been reduced by manufacturers. The BPB can be considered an initial step to studying rubber allergy, but it does not seem to be sufficient, according to the data of the present work. The adoption of a specific supplementary series, in addition to testing with boot and glove fragments, is recommended for better diagnostic accuracy.^2^, ^8^, ^9^

## Financial support

None declared.

## Authors' contributions

Vanessa Barreto Rocha: design and planning of the study; data collection; drafting and editing of the manuscript; or critical review of important intellectual content; collection, analysis, and interpretation of data; intellectual participation in the propaedeutic and/or therapeutic conduct of the studied cases; approval of the final version of the manuscript.

Erica Possa de Abreu: design and planning of the study; data collection, or analysis and interpretation of data; drafting and editing of the manuscript or critical review of important intellectual content; collection, analysis, and interpretation of data; critical review of the literature; approval of the final version of the manuscript.

Maria Antonieta Rios Scherrer: Design and planning of the study; data collection, or analysis and interpretation of data; statistical analysis; drafting and editing of the manuscript; or critical review of important intellectual content; collection, analysis, and interpretation of data; effective participation in research orientation; intellectual participation in the propaedeutic and/or therapeutic conduct of the studied cases; critical review of the literature; approval of the final version of the manuscript.

## Conflicts of interest

None declared.
